# Quantitative Chest CT Analysis: Three Different Approaches to Quantify the Burden of Viral Interstitial Pneumonia Using COVID-19 as a Paradigm

**DOI:** 10.3390/jcm13237308

**Published:** 2024-12-01

**Authors:** Salvatore Claudio Fanni, Leonardo Colligiani, Federica Volpi, Lisa Novaria, Michele Tonerini, Chiara Airoldi, Dario Plataroti, Brian J. Bartholmai, Annalisa De Liperi, Emanuele Neri, Chiara Romei

**Affiliations:** 1 Department of Translational Research, Academic Radiology, University of Pisa, 56126 Pisa, Italy; fannisalvatoreclaudio@gmail.com (S.C.F.); leonardocolligiani@gmail.com (L.C.); federica.93.volpi@gmail.com (F.V.); dario.plataroti@gmail.com (D.P.); emanuele.neri@unipi.it (E.N.); 2 2nd Radiology Unit, Department of Diagnostic Imaging, Pisa University-Hospital, Via Paradisa 2, 56100 Pisa, Italy; l.novaria@ao-pisa.toscana.it (L.N.); a.deliperi@ao-pisa.toscana.it (A.D.L.); 3 Department of Emergency Radiology, Pisa University-Hospital, Via Paradisa 2, 56100 Pisa, Italy; m.tonerini@tiscali.it; 4 Department of Translational Medicine, University of Eastern Piemonte, 13100 Novara, Italy; chiara.airoldi@uniupo.it; 5 Division of Radiology, Mayo Clinic, Rochester, MN 85259, USA; bartholmai.brian@mayo.edu

**Keywords:** CT quantitative, thorax, texture analysis, pneumonia, viral, COVID-19

## Abstract

**Objectives:** To investigate the relationship between COVID-19 pneumonia outcomes and three chest CT analysis approaches. **Methods:** Patients with COVID-19 pneumonia who underwent chest CT were included and divided into survivors/non-survivors and intubated/not-intubated. Chest CTs were analyzed through a (1) Total Severity Score visually quantified by an emergency (TSS1) and a thoracic radiologist (TSS2); (2) density mask technique quantifying normal parenchyma (DM_Norm 1) and ground glass opacities (DM_GGO1) repeated after the manual delineation of consolidations (DM_Norm2, DM_GGO2, DM_Consolidation); (3) texture analysis quantifying normal parenchyma (TA_Norm) and interstitial lung disease (TA_ILD). Association with outcomes was assessed through Chi-square and the Mann–Whitney test. The TSS inter-reader variability was assessed through intraclass correlation coefficient (ICC) and Bland–Altman analysis. The relationship between quantitative variables and outcomes was investigated through multivariate logistic regression analysis. Variables correlation was investigated using Spearman analysis. **Results:** Overall, 192 patients (mean age, 66.8 ± 15.4 years) were included. TSS was significantly higher in intubated patients but only TSS1 in survivors. TSS presented an ICC of 0.83 (0.76; 0.88) and a bias (LOA) of 1.55 (−4.69, 7.78). DM_Consolidation showed the greatest median difference between survivors/not survivors (*p* = 0.002). The strongest independent predictor for mortality was DM_Consolidation (AUC 0.688), while the strongest independent predictor for the intensity of care was TSS2 (0.7498). DM_Norm 2 was the singular feature independently associated with both the outcomes. DM_GGO1 strongly correlated with TA_ILD (ρ = 0.977). **Conclusions:** The DM technique and TA achieved consistent measurements and a better correlation with patient outcomes.

## 1. Introduction

Many different viruses, such as influenzas, rhinovirus, adenovirus and SARS-CoV-2 can determine lower respiratory tract infections and viral pneumonia, increasing hospitalizations and mortality [1,2]. Chest CT is the preferred method for disease diagnosis, although it shows heterogeneous and nonspecific findings detectable even in nonviral pathologies, such as interstitial lung disease (ILD). These findings include ground-glass opacities (GGOs), interlobular septal thickening, “crazy paving” pattern, consolidation with air bronchogram, and less commonly bronchiectasis, reversed halo-sign and lymphadenopathy [1,2,3,4,5].

With the SARS-CoV-2 pandemic, CT has gained importance in severity assessment of coronavirus disease 2019 (COVID-19) [6,7,8,9]. However, severity evaluation largely relied on a qualitative assessment, which suffers from inter- and intra-observer variability and limited sensitivity in detecting subtle findings [10,11,12]. Moreover, qualitative assessment does not provide quantitative prognostic or predictive data [3,10,11,12]. Over the years, several different quantitative analysis tools have shown promising results in supporting the diagnosis, prognosis and longitudinal assessment of diffuse lung disease, which are characterized by many overlapping radiological findings that may be subject to different interpretations on a visual assessment [13]. Quantitative CT includes a wide variety of techniques, such as visual quantification, the density mask (DM) technique, and texture analysis. In the former, radiologists assign scores based on lung involvement; in the DM technique, each voxel is represented with different colors according to its Hounsfield unit density using pre-established thresholds; texture analysis classifies and quantifies lung patterns according to the voxel texture [14]. In the past few years, the relationship between quantitative CT outputs and COVID-19 pneumonia severity have been widely investigated [2,9,15,16]. However, how these outputs are associated with patients’ outcomes and how they correlate with each other is still underexplored. This study aims to describe the relationship between COVID-19 pneumonia outcomes and chest CT quantitative outputs using three different approaches: Total Severity Score (TSS), a DM technique and texture analysis. Secondly, the correlation between quantitative CT outputs was evaluated.

## 2. Materials and Methods

### 2.1. Case Selection

All consecutive patients with COVID-19 hospitalized in Pisa University Hospital, between March and April 2020 were eligible for this retrospective study. Inclusion criteria comprised proven infection of SARS-CoV-2 on a positive RT-PCR test within a week from CT acquisition. Patients with severe motion artifacts at CT and unavailable RT-PCR were excluded. Patients were divided according to the mortality (survivors, non-survivors) and to the required intensity of care (not intubated, intubated).

### 2.2. CT Acquisition

All volumetric non-contrast chest CT scans were performed on supine patients in the emergency department using a 40-slice Siemens Somatom Sensation scanner (Siemens Healthineers, Erlangen, Germany) and a 64-slice General Electric Light Speed scanner (General Electric Co., Boston, MA, USA). The acquisition parameters were 120 kV, 284 mAs, 1.84 spiral pitch factor, 0.625 collimation width, 512 × 512 matrix, 1.5 reconstruction thickness, B31 kernel and 120 kV, 169 mAs, 0.98 spiral pitch factor, 0.625 collimation width, 512 × 512 matrix, 1.25 reconstruction thickness, and standard kernels for the 40-slice and 64-slice scanners, respectively.

### 2.3. Total Severity Score

Using the TSS, a subjective quantitative evaluation was independently performed by an emergency radiologist (TSS1) and by a thoracic radiologist (TSS2) [17]. A score (range 0–4) was assigned to each lung lobe to represent the percentage of parenchymal involvement: none (0%), minimal (1–25%), mild (26–50%), moderate (51–75%), or severe (76–100%). The TSS was determined by summing the scores of the five lobes (range 0–20).

### 2.4. Density Mask Technique

**Figure 1:**
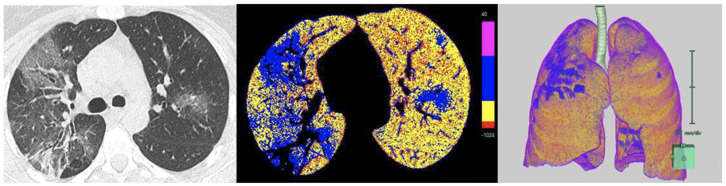
Density mask analysis of CT scan in a 46-year-old female patient. From left to right: axial CT slice with multiple bilateral ground glass opacities, the color image overlays (red = emphysema, yellow = normal parenchyma, blue = ground-glass opacities (GGOs), pink = consolidations) and the 3D volume rendering reconstruction.

CT scans were quantitatively analyzed using a DM technique through the Synapse 3D software (Vrs 6.4, Fujifilm, Ratingen, Germany). The Lung Analysis tool in the software performed a semi-automated lung segmentation with predefined thresholds and allowed the definition of DM based on the distribution of different values of Hounsfield Unit (HU) (Figure 1).

A specific densitometric threshold model (Pisa Model) was created based on thresholds published in previous studies. In particular, the thresholds for emphysema (−1024 to −950 HU), normal parenchyma (−949 to −750 HU) and GGO (−749 to −300 HU) were adapted from Shin et al., while for consolidations, the thresholds (−299 to +40 HU) were adapted from Cheng et al. [18,19]. The percentage of GGO and normal lung in the total lung volume was calculated and defined as DM_GGO1 and DM_Norm1. However, the Fujifilm Lung Analysis Airway software’s (Vrs 6.4) semi-automated segmentation did not include much of the consolidated parenchyma, resulting in a loss of this pattern and underestimation of the total lung volume. Therefore, a second computation was performed after the consolidation’s manual segmentation, which resulted in the calculation of the percentage of consolidated parenchyma and new values of GGO and normal lung percentages. These parameters were defined as DM_GGO2, DM_Norm2, and DM_Consolidation. The time required for each manual segmentation of consolidations was recorded.

### 2.5. Texture Analysis

**Figure 2:**
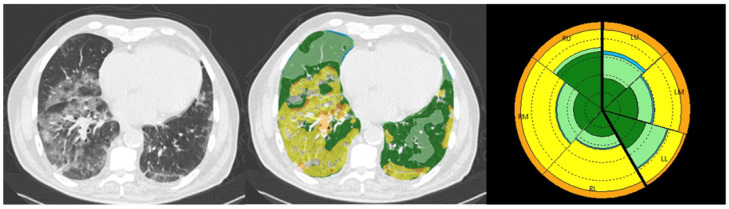
CALIPER analysis of a CT scan in a 73-year-old male patient. From left to right: axial CT slice, axial CALIPER-derived color image overlays (dark and light green = norm, yellow = GG, orange = Ret), the glyph of the lung parenchymal patterns.

Finally, a texture analysis was carried out using CALIPER (Vrs 1.0), a software developed by the Biomedical Imaging Resource, Mayo Clinic, Rochester, MN, USA, to quantify 7 lung parenchymal patterns: severe, moderate, and mild–lower attenuation (emphysema), normal parenchyma, GGO, reticular opacities and honeycombing (Figure 2).

For each pattern, the software computed the percentage of total lung volume. The sum of GGO, reticular opacities and honeycombing percentages was defined as Interstitial Lung Disease (ILD) percentage [20,21]. In this study, the percentage of normal parenchyma and ILD were assessed and defined as TA _Norm and TA _ILD.

### 2.6. Statistical Analysis

The clinical variables were described using absolute and relative frequencies for categorical variables and the mean and standard deviation or median and interquartile range for quantitative ones. The association between the variables and outcomes was assessed using an unpaired Student’s *t*-test or the Mann–Whitney as appropriate. The intraclass correlation coefficient (ICC) was calculated to assess the inter-reader variability of TSS, while a Bland–Altman plot was used to represent the difference scores of two measurements against the mean for each subject, and bias and limits of agreement (LOAs) were calculated. To investigate the relationship between quantitative variables and outcomes, a univariate logistic regression analysis was performed, which was followed by a multivariate analysis adjusted for age and sex. The association between quantitative variables was investigated through Spearman correlation analysis and described as very weak (0 < ρ ≤ 0.19), weak (0.20 < ρ ≤ 0.39), moderate (0.40 < ρ≤ 0.59), strong (0.60 < ρ ≤ 0.79) and very strong (0.80 < ρ≤ 1). *p*-values of less than 0.05 (two-tailed) were considered statistically significant. Statistical analyses were conducted using with Stata 15 (StataCorp 2017, College Station, TX, USA) and SAS 9.4.

## 3. Results

The final cohort included 192 patients (mean age 66.8 ± 15.4 years), 72 females (37.5%) and 120 males (62.5%). Thirteen-four patients (17.7%) died during the hospitalization. There was a statistically significant difference in age distribution between survivors (65.6 ± 15) and non-survivors (72.6 ± 16), but not in sex distribution. Fifty-seven patients (29.7%) required intubation. There was a significant difference in age distribution (*p* < 0.05) between not intubated (68.7 ± 16) and intubated (62.6 ± 13), and in sex distribution (*p* < 0.001), with 46% females and 54% males in the former group of patients and 17.5% females and 82.5% males in the latter. A significant difference was demonstrated for both TSS2 (*p* < 0.001) and TSS1 (*p* = 0.002) between intubated and not intubated patients, while regarding the mortality outcome, a significant difference was demonstrated only for TSS1 (*p* = 0.017) (Table 1).

**Figure 3:**
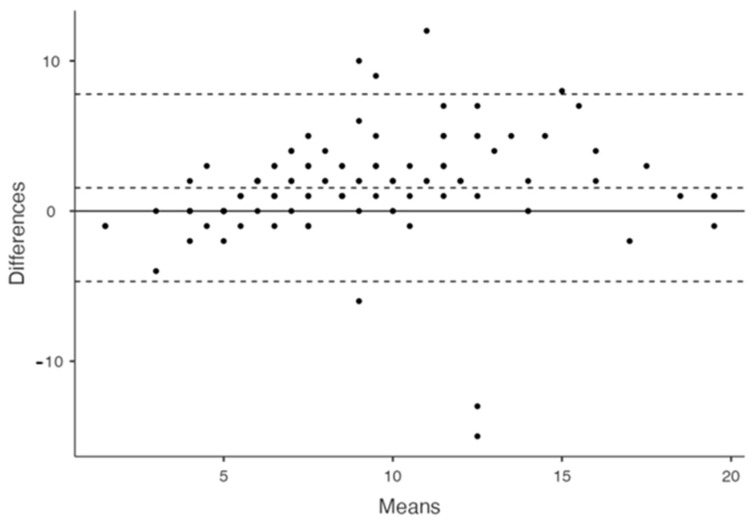
Bland–Altman plot of TSS1 and TSS2.

There was a strong agreement between the two readers with an ICC (95% CI) value of 0.83 (0.76; 0.88). The Bland–Altman plot showed a bias (LOA) of 1.55 (−4.69, 7.78), and that some observations were quite discordant as they were outside the LOA (Figure 3).

The first outputs of the DM technique were DM _GGO1 and DM _Norm1, which showed a statistically significant median difference between the two outcome subgroups, particularly DM_Norm1 between not intubated and intubated patients (*p* = 0.008). After consolidations, manual segmentation, requiring a mean of 4 min (range 1–24), the strongest median difference was demonstrated for DM_Consolidation between survivors and non survivors (*p* = 0.002). A significant difference was shown for DM _GGO2 between care intensity subgroups (*p* = 0.006) with no such difference between survivors and non-survivors (*p* = 0.181).

A statistically significant difference was demonstrated for TA _ILD and TA _Norm for both outcome subgroups: the strongest one for TA _Norm (*p* = 0.003) between survivors and non-survivors (Table 2).

Univariate and multivariate logistic regression analysis results are resumed in Table 3 and Table 4.

At multivariate analysis, the strongest independent predictor for mortality was DM_Consolidation (AUC 0.688), which was followed by DM_Norm2 (0.6815) and TA_Norm (0.6882). The strongest independent predictor for required intensity of care was TSS2 (0.7498), which was followed by TSS1 (0.7342), DM_Norm 1 (0.7337) and DM_Norm 2 (0.7283). Spearman correlation analysis revealed a significant (*p* < 0.001) moderate to strong correlation between TSS and quantitative analysis outputs and a strong to very strong correlation between DM and TA outputs (Table 5).

The strongest positive correlation was found between DM_GGO1 and TA_ILD (0.977), while the strongest negative correlation was found between DM_GGO1 and TA_Norm (−0.927).

## 4. Discussion

In this study, different chest CT analysis quantitative outputs were found to be variably associated with mortality and the required intensity of care. In particular, the consolidations percentage measured through DM techniques was found to be the strongest independent predictor for mortality, while TSS2 for the required intensity of care. Secondly, a strong correlation was found between DM technique and texture analysis, while both were moderately correlated with TSS.

TSS was the first quantitative approach adopted in this study. A satisfactory ICC of 0.83 was found between the two raters, which was slightly lower then Elmokadem et al. [22], who reported an ICC of 0.99 among three raters [23]. However, a bias toward higher scores was observed in TSS1 as compared to TSS2, which was possibly due to the different fields of expertise [23]. This difference may explain the different relationships between ratings and outcomes. Indeed, TSS1 was better correlated with the mortality outcome, while TSS2 was demonstrated the strongest in the [7,24] dependent predictor for the required intensity of care. According to Zakariee et al., the radiologist experiences did not significantly affect the prognostic value of CT severity scores to predict mortality in COVID-19 patients [25]. However, field expertise seems to have some effect, which may pose limitations for visual CT scores as well as time consumption and threshold discrepancies [7,24].

The DM technique constituted the second quantitative approach adopted in this study. The DM technique outputs were found to be significantly associated with the assessed outcomes also without manual segmentation of consolidation. Yet, including manually segmented consolidations nullified the difference in GGO percentage between survivors and non-survivors. This could be attributed to the higher proportion of consolidations in severely ill patients, which probably leads to poorer outcomes, resulting in increased total lung volume and relatively lower GGO volume in this subgroup. Consolidation volume alone was demonstrated to be the strongest independent predictor for mortality. Nonetheless, the singular quantitative feature independently linked to both survival and the required intensity of care was DM_Norm 2. This result highlights that an objective quantification of healthy parenchyma obtained through a relatively simple technique may provide additional insights concerning the patient’s prognosis compared to other quantitative parameters. Additionally, among the objective quantitative features, DM_Norm 1 emerged as the most robust predictor for the intensity of care, being free from subjective bias and requiring minimal time investment.

A similar DM technique approach was adopted by Lanza et al., who used an interval between −500 and 100 HU to identify the compromised lung volume, which resulted in the most accurate risk factor for in-hospital mortality and intubation [26].

A different interval (−600–250 HU) was adopted by Kang et al., including both GGO and consolidations [27]. The authors showed that adding this DM technique parameter to a decision tree, compared with clinical variables alone, improved its accuracy [27]. There is a significant heterogeneity in the literature in defining the thresholds used for the DM technique. Indeed, the choice of how many thresholds and which thresholds to adopt is arbitrary, and it is difficult to determine the optimal ones. This issue might be addressed through texture analysis, albeit with the significant trade-off of heightened complexity and reduced accessibility.

CALIPER software has been widely used with promising results for the quantitative analysis of ILDs [20,28], and it could be also used to evaluate viral interstitial pneumonia, specifically COVID-19, as these diseases share GGOs and reticulations patterns. This study found significant differences in percentages for both TA_ILD and TA_Norm in all the subgroups, with higher TA_ILD in patients with adverse outcomes, consistently with Romei et al. [29]. However, in multivariate analysis, only TA_Norm was identified as independently associated, and solely with survival. The assessment of healthy parenchyma has once again proven to be more informative than the evaluation of disease burden.

The strongest correlation was found between the two quantitative methods, particularly between TA_ILD and DM_GGO1. This result is explained by the fact that both the first computing of DM technique and CALIPER did not take consolidations into account. Similarly, Yousef et al. investigated the correlation between a semi-quantitative score and the percentage of compromised lung volume, which was computed by using the same software and thresholds and summing up GGO and consolidation percentages. The authors reported a higher Spearman’s correlation coefficient of 0.934. A potential explanation is that the authors compared the percentage of the summed-up compromised lung volume and the averaged mean visual scores of two raters [30].

This study has several limitations: small sample size from one institution; the cohort included only COVID-19 patients from the first pandemic wave, which was characterized by higher severity compared to subsequent waves [31]; and the typical patterns of COVID-19 pneumonia that were seen during the early waves of the pandemic are now rarely encountered due to new variants and vaccination, so the association between the analyzed outputs and outcomes should be re-examined in these new settings. However, the global landscape of viral infections has demonstrated in recent years its ability to rapidly evolve, and developing robust and objective chest CT quantitative approaches has demonstrated to be of paramount importance in managing patients with viral interstitial pneumonia. Another significant limitation is the lack of clinical variables in this study. A multivariable analysis model that also included clinical variables might have yielded more effective results. We plan to explore this aspect further in future studies. Finally, this study examined only three quantitative approaches, which may limit its scope, particularly in a field that is evolving rapidly [32,33,34,35].

## 5. Conclusions

To our knowledge, this is the first study comparing three different quantitative strategies in the same cohort. The three quantitative approaches were demonstrated to be variably associated with intensity of care and mortality. At multivariate analysis, both TSS and DM_Norm 1 were found to be associated with the required intensity of care. Conversely, DM_Cons and the percentage of healthy parenchyma, regardless of the approach, served as prognostic indicators for survival.

Nonetheless, the singular quantitative feature independently associated with both survival and the necessary intensity of care was DM_Norm 2. In this context, the DM technique may serve as a beneficial compromise between visual quantification, which yielded inconsistent results at a more thorough analysis, and more complex quantitative methods, such as texture analysis, which are less accessible. Given the similarities of CT findings and considering COVID-19 pneumonia as a paradigm, these results may be deemed transposable for the assessment of other viral interstitial pneumonia.

## Figures and Tables

**Table 1:** Age, sex and TSS1 and TSS2 [IQR] distribution in different groups.

	Mortality			Intensity of Care		
	Survivors (*n* = 158)	Non Survivors (*n* = 34)	*p* Value	Not Intubated (*n* = 135)	Intubated (*n*= 57)	*p* Value
TSS1 *	9 [5; 12]	11 [7; 15.80]	0.017	8 [5; 12]	11 [7; 14]	0.002
TSS2 *	6.5 [5; 10]	8 [5; 11]	0.117	6 [5; 9]	9 [6; 11]	<0.001
Age	65.6 ± 15	72.6 ± 16	<0.001	68.7 ± 16	62.6 ± 13	0.003
Sex (M)	60.7%	70.6%	0.283	54%	82.5%	<0.001

* TSS Total Severity Score.

**Table 2:** Median (IQR) of CT parameters for each group calculated through density mask technique and texture analysis.

	Mortality			Intensity of Care		
	Survivors	Non-Survivors	*p* Value	Not Intubated	Intubated	*p* Value
DM _GGO1 *	30.70 [19.92; 45.05]	39.40 [24.50; 54.80]	0.047	30.50 [19.20; 44.85]	38.60 [24.20; 54.60]	0.020
DM _Norm1 *	64.30 [52.82; 77.95]	57.80 [42.98; 70.43]	0.039	65.80 [52.95; 78.50]	57.00 [43.90; 74.60]	0.015
DM _GGO2 *	30.20 [19.63; 43.85]	36.70 [23.28; 46.93]	0.182	30.20 [18.60; 42.85]	39.10 [25.00; 52.70]	0.006
DM _Norm2 *	63.55 [50.23; 76.30]	54.65 [34.30; 68.52]	0.010	63.30 [49.75; 77.05]	55.20 [37.80; 72.20]	0.023
DM _Consolidation *	2.5 [1.10; 5.72]	7.3 [2.0; 13.30]	0.002	2.50 [1.00; 6.85]	3.70 [1.50; 7.00]	0.047
TA _ILD *	17.98 [7.31; 33.46]	30.06 [13.91; 48.81]	0.013	18.21 [6.46; 35.02]	27.71 [11.46; 46.58]	0.032
TA_Norm *	76.60 [59.31; 85.50]	61.42 [41.34; 74.84]	0.003	75.47 [58.38; 85.90]	64.12 [45.25; 83.44]	0.033

* Density mask technique (DM) and texture analysis (TA) outputs are calculated as percentages of the whole lung volume. DM_GGO1: ground glass opacities percentage calculated before manual segmentation of consolidations. DM_Norm1: normal parenchyma percentage calculated before manual segmentation of consolidations. DM_GGO2: ground glass opacities percentage calculated after manual segmentation of consolidations. DM_Norm2: normal parenchyma percentage calculated after manual segmentation of consolidations. DM_Consolidation: consolidated parenchyma percentage. TA_ILD: interstitial lung disease percentage. TA_Norm: normal parenchyma percentage.

**Table 3:** Univariate and multivariate analysis adjusted for age and sex of risk factors considered associated with survival.

Risk Factor	Univariate Model		Multivariate Model	
	AUC	OR (95% CI)	AUC	OR (95% CI)
TSS1 *	0.6309	1.10 (1.02–1.91)	0.6759	1.08 (1.00–1.17)
TSS2 *	0.5851	1.08 (0.99–1.17)		
DM_GGO1 **	0.6073	1.01 (0.99–1.03)		
DM_Norm1 **	0.6133	0.98 (0.96–1.00)		
DM_GGO2 **	0.5731	1.01 (0.99–1.03)		
DM_Norm2 **	0.6413	0.98 (0.96–0.99)	0.6815	0.97 (0.96–0.99)
DM_Consolidation2 **	0.6653	1.07 (1.02–1.12)	0.6888	1.08 (1.04–1.14)
TA_ILD **	0.6363	1.02 (1.00–1.03)		
TA_Norm **	0.6646	0.97 (0.96–0.99)	0.6882	0.98 (0.96–0.99)
Age	0.6557	1.03 (1.00–1.06)		
Sex	0.5491	1.55 (0.69–3.46)		

* TSS Total Severity Score. ** Density mask technique (DM) and texture analysis (TA) outputs are calculated as percentages of the whole lung volume. DM_GGO1: ground glass opacities percentage calculated before manual segmentation of consolidations. DM_Norm1: normal parenchyma percentage calculated before manual segmentation of consolidations. DM_GGO2: ground glass opacities percentage calculated after manual segmentation of consolidations. DM_Norm2 Normal parenchyma percentage calculated after manual segmentation of consolidations. DM_Consolidation: consolidated parenchyma percentage. TA_ILD: interstitial lung disease percentage. TA_Norm: normal parenchyma percentage.

**Table 4:** Univariate and multivariate analysis adjusted for age and sex of risk factors considered associated with required intensity of care.

Risk Factor	Univariate Model		Multivariate Model	
	AUC	OR (95%CI)	AUC	OR (95% CI)
TSS1 *	0.6383	1.09 (1.02–1.16)	0.7342	1.11 (1.04–1.19)
TSS2 *	0.6787	1.14 (1.06–1.23)	0.7498	1.17 (1.07–1.27)
DM_GGO1 **	0.6067	1.01 (1.00–1.03)		
DM_Norm1 **	0.6114	0.98 (0.96–0.99)	0.7337	0.97 (0.95–0.99)
DM_GGO2 **	0.6254	1.02 (1.00–1.05)		
DM_Norm2 **	0.6043	0.98 (0.97–0.99)	0.7283	0.97 (0.96–0.99)
DM_Consolidation2 **	0.5906	1.00 (0.96–1.04)		
TA_ILD **	0.5981	1.01 (0.99–1.02)		
TA_Norm **	0.5975	0.98 (0.97–1.01)		
Age	0.6305	0.97 (0.95–0.99)		
Sex	0.6419	3.99 (1.86–8.51)		

* TSS Total Severity Score. ** Density mask technique (DM) and texture analysis (TA) outputs are calculated as percentages of the whole lung volume. DM_GGO1: ground glass opacities percentage calculated before manual segmentation of consolidations. DM_Norm1: normal parenchyma percentage calculated before manual segmentation of consolidations. DM_GGO2: ground glass opacities percentage calculated after manual segmentation of consolidations. DM_Norm2: normal parenchyma percentage calculated after manual segmentation of consolidations. DM_Consolidation: consolidated parenchyma percentage. TA_ILD: interstitial lung disease percentage. TA_Norm: normal parenchyma percentage.

**Table 5:** Spearman correlation analysis results.

	DM _GGO1 *	DM _Norm1 *	DM _GGO2 *	DM _Norm2 *	DM _Consolidation *	TA _ILD *	TA_Norm *	TSS 1 **	TSS 2 **
DM _GGO1 *	-								
DM _Norm1 *	−0.967	-							
DM _GGO2 *	0.967	−0.930	-						
DM _Norm2 *	−0.940	0.954	−0.947	-					
DM _Consolidation *	0.745	−0.717	0.719	−0.804	-				
TA _ILD *	0.977	−0.950	0.938	−0.946	0.780	-			
TA_Norm *	−0.927	0.923	−0.883	0.920	−0.770	−0.965	-		
TSS 1 **	0.740	−0.735	0.708	−0.757	0.699	0.755	−0.767	-	
TSS 2 **	0.644	−0.656	0.620	−0.676	0.577	0.677	−0.683	0.781	-

* Density mask technique (DM) and texture analysis (TA) outputs are calculated as percentages of the whole lung volume. DM_GGO1: ground glass opacities percentage calculated before manual segmentation of consolidations. DM_Norm1: normal parenchyma percentage calculated before manual segmentation of consolidations. DM_GGO2: ground glass opacities percentage calculated after manual segmentation of consolidations. DM_Norm2: normal parenchyma percentage calculated after manual segmentation of consolidations. DM_Consolidation: consolidated parenchyma percentage. TA_ILD: interstitial lung disease percentage. TA_Norm: normal parenchyma percentage. ** TSS Total Severity Score.

## Data Availability

The original contributions presented in the study are included in the article, further inquiries can be directed to the corresponding author.
